# Improving the Admission Clerking Process and Documentation Completion in an Adult Acute Inpatient Psychiatric Unit

**DOI:** 10.1192/bjo.2026.11398

**Published:** 2026-06-30

**Authors:** Fazila Imtiaz, Asad Ahmad, Divyesh Dholariya

**Affiliations:** East London Foundation Trust, Luton, United Kingdom

## Abstract

**Aims:**

Timely and accurate admission clerking is essential for safe and effective care in inpatient psychiatric settings. In our adult acute inpatient psychiatric unit, admission clerking was frequently delayed and inconsistently completed, increasing risks to patient safety, delaying treatment initiation, and impairing multidisciplinary communication. This quality improvement (QI) project aimed to improve the timeliness and completeness of admission clerking by ensuring completion within 72 hours of admission and achieving at least 70% documentation completeness by December 2025.

**Methods:**

A QI methodology using Plan–Do–Study–Act (PDSA) cycles was employed. Afishbone diagram and driver diagram were developed to identify contributory factors and guide interventions. Baseline data were collected to assess current performance and identify priority areas for improvement. Two change ideas were tested. The first involved extending the target timeframe for admission clerking completion to within 72 hours of admission, which was clearly communicated to all medical staff. The second involved introducing a standardized admission clerking template to ensure consistent documentation of key domains, including mental state examination, risk assessment, physical health, and social history. Data on timeliness and completeness of admission clerking were collected and reviewed across sequential PDSA cycles.

**Results:**

Following implementation of the first intervention, the initial PDSA cycle demonstrated a significant qualitative improvement in admission clerking completion rates, although quantitative variability in documentation quality remained. During the second PDSA cycle, the extended 72-hour target was retained and combined with the standardized clerking template. Over the final twelve weeks of data collection, this combined approach resulted in a sustained improvement in both timeliness and completeness, achieving a 100% admission clerking completion rate.

**Conclusion:**

Clearly defined timeframes combined with standardized documentation tools can significantly improve the quality, timeliness, and sustainability of admission clerking in inpatient psychiatric settings. Plans are underway to scale this intervention across the wider trust and continue data collection to evaluate long-term impact and sustainability.

